# Ethical Artificial Intelligence for Digital Health Organizations

**DOI:** 10.7759/cureus.7202

**Published:** 2020-03-07

**Authors:** Angela Joerin, Michiel Rauws, Russell Fulmer, Valerie Black

**Affiliations:** 1 Psychology, X2AI Inc., San Francisco, USA; 2 CEO & Founder, X2AI Inc., San Francisco, USA; 3 Counseling, Northwestern University, Evanston, USA; 4 Anthropology, University of California, Berkeley, USA

**Keywords:** artificial intelligence, ethics, codes of ethics, mental health, healthcare technology, chatbot

## Abstract

This technical report describes the methods undertaken by a US-based Digital Health company (X2AI or X2 for short) to develop an ethical code for startup environments and other organizations delivering emotional artificial intelligence (AI) services, especially for mental health support. With a growing demand worldwide for scalable, affordable, and accessible health care solutions, the use of AI offers tremendous potential to improve emotional well-being. To realize this potential, it is imperative that AI service providers prioritize clear and consistent ethical guidelines that align with global considerations regarding user safety and privacy. This report offers a template for an ethical code that can be implemented by other emotional AI services and their affiliates. It includes practical guidelines for integrating support from clients, collaborators, and research partners. It also shows how existing ethical systems can inform the development of AI ethics.

## Introduction

Chatbots are one of the most widely adopted iterations of artificial intelligence (AI), as is the idea of creating a chatbot for therapeutic dialog [1-2]. But, when combined with today’s ever-advancing natural language processing (NLP) and other modes of AI that make more sensitive communication with human users possible, emotionally supportive chatbots are anything but retrograde.

X2 is a company that creates customized chatbots (AI coaches) for an array of use-cases, most of which focus on exploring and uplifting emotional well-being. This technology is highly scalable, easy to use, available on demand, and swiftly adaptable across languages, cultures, and other important contexts. This means that a supportive AI coach can complement conventional mental health care and even reach users in times and places where other modes of care cannot. However, to achieve this potential, it is imperative that members of this industry adopt clear and consistent ethical practices for AI pertaining to human emotions. We aim to offer an ethical code to serve as a template for startups and other organizations which provide emotional AI services, especially within the context of mental health support.

X2’s starting place for developing such an ethical code was the realization that for emotional support AI, it is important to consider emerging AI ethics alongside established mental health professional guidelines. We began by asking: “How and where do these ethical approaches converge?”

## Technical report

Establishing an ethical code for emotional artificial intelligence

**Figure 1:**
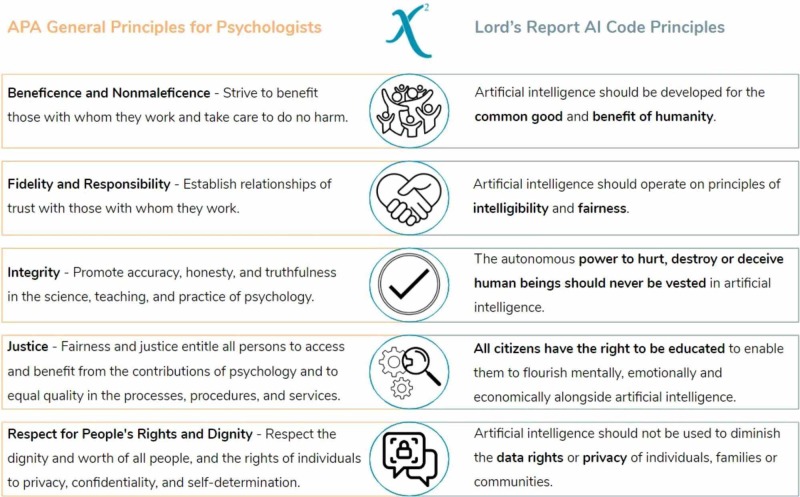
The intersection of fundamental ethical systems

The X2 ethical code’s foundation is an overlap observed between the American Psychological Association’s (APA) General Principles for Psychologists and the UK Lord’s Report AI Code Principles (Figure 1) [3-4].

**Figure 2:**
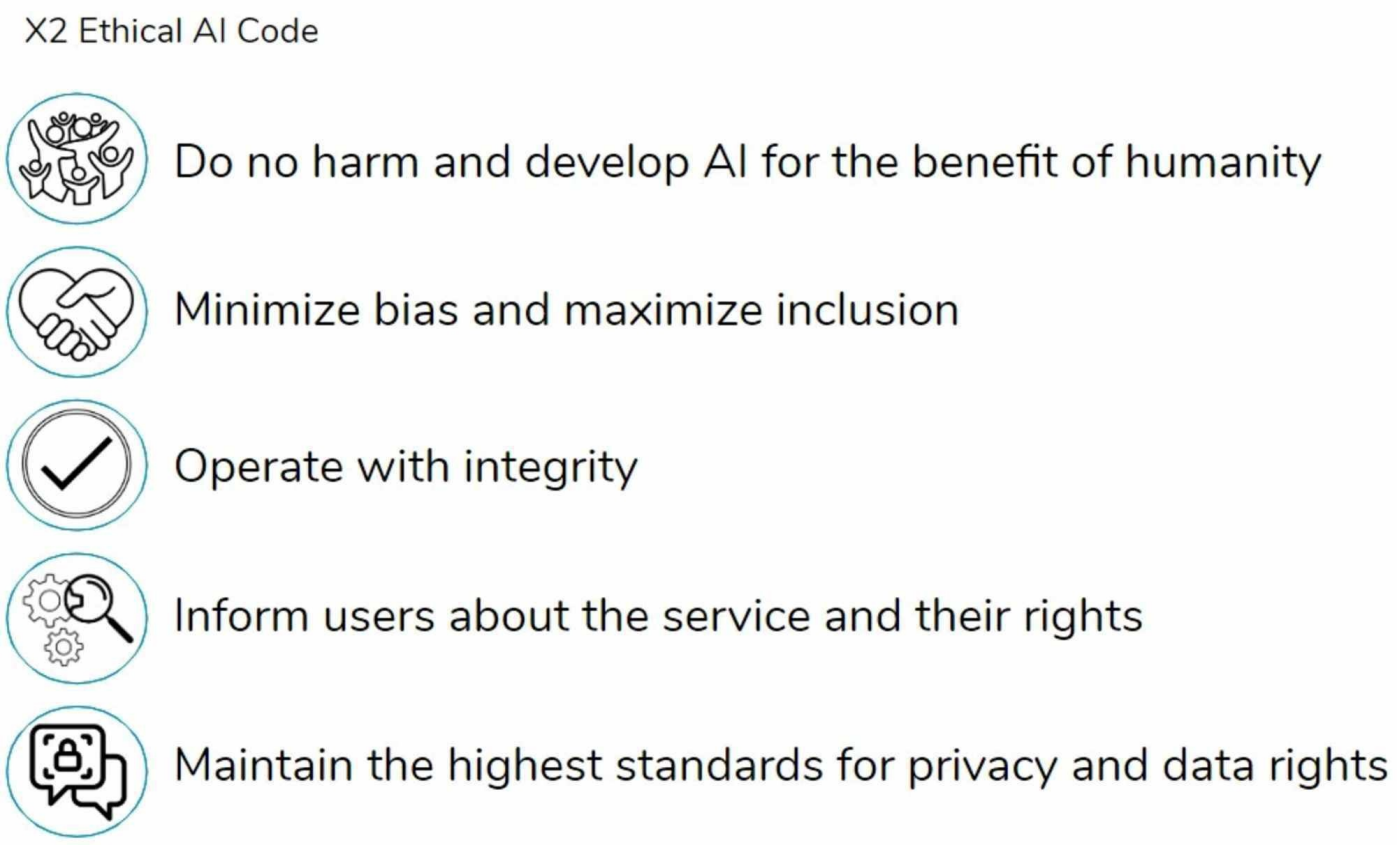
X2 ethical AI code AI, artificial intelligence

The aim of integrating these fields is to produce a robust ethical code that adapts as emotional AI advances. X2 persistently applies these foundational ethical principles to the company’s procedures for creating and maintaining the AI system. This approach aims to further ongoing discussion of AI ethics by providing a practical example of how an ethical AI code can emerge through detecting and deepening the link between different ethical systems. Figure 2 displays our resulting ethical AI code after merging principles from the APA’s General Principles for Psychologists and the UK Lord’s Report AI Code Principles.

This ethical code for emotional AI is designed to protect the well-being of all users across the development, delivery, and maintenance of a startup or other digital health organization’s AI services. X2’s strategy for operationalizing this code consists of the following four key components: 

1. Core ethical principles and sources.

2. Privacy and security measures.

3. Team expertise and training.

4. Research and development.

Core ethical principles and sources

X2’s ethical guiding principles arise from the intersection of mental health care and technology ethics: we refuse to pursue or deliver services, whether independently or in collaboration with other organizations, that are likely to cause harm. Aligning with Google’s 2018 AI principles, X2 commits to blocking any use of products and services, in which they would be part of the implementation of any weapons or other technologies whose purpose is to cause injury to people [5]. X2 identifies commitment to harm reduction as a guiding principle for the implementation of emotional AI.

The principles recommended by Luxton et al., who comprehensively examine the ethical challenges of applying AI technologies to mental health care, further inform how we monitor and secure user privacy, safety, autonomy, and trust [6]. X2’s most important method for sustaining this intersection of mental health care and technology ethics is seeking expert guidance on an ongoing basis. X2 is further motivated by the White House’s report on preparing for the future of AI, which calls for organizational strategies that focus on international, industry-led engagement [7].

To realize this aim, X2 regularly seeks guidance from industry and academic experts, inviting critical and reflective thinking on company practices, both established and developing. We maintain a standing medical ethical board consisting of faculty from academic institutions. The chairwoman of this advisory board also holds an observer seat on the board of directors of X2 and thereby can serve as a liaison for the interests of this advisory board. X2 also routinely reaches out to other experts for ethical guidance when implementing new technologies or developing new content such as expert research partners from Duke, Northwestern, and Stanford Universities. Likewise, when launching a service customized for a particular geographic region, X2 seeks local experts to evaluate and guide efforts at every step. Like any of the partnering organizations, the experts with whom X2 engages must demonstrate a clear commitment to harm reduction. By making this a regular component of developing and maintaining AI services, digital health organizations create more opportunities to link ethical principles to concrete practices.

The following sections describe policies and procedures developed by X2 to support and advance the companies ethical commitments.

Privacy and security measures

Ensuring the rights of users to confidentiality, privacy, and control over how their data are handled is of utmost importance. Without earning the trust and confidence of users, AI for emotional support will not achieve its potential to help people. In particular, principles of transparency and accountability guide company practices.

Transparency

Keeping pace with quickly evolving international data privacy standards, including the European Union General Data Protection Regulation (GDPR), is essential [8]. X2 users have the same data privacy rights regardless of the location or other demographic considerations. The company fully complies with the standards outlined by the GDPR and maintains these standards for all users worldwide. X2 also offers Health Insurance Portability and Accountability Act (HIPAA)-compliant AI coaching services for integration with the existing electronic health record systems, and HIPAA-compliant fully encrypted messaging platforms.

All X2 users are presented with the Terms of Use and Privacy Policy at the start of their first AI coach interaction. The privacy policy is customized based on the agreements with each individual customer. Through these custom privacy policies, X2 can go as far as to delete any user data after each interaction. The privacy policies use clear, non-expert language to describe how we manage user data. Importantly, the business model enables X2 to entirely forgo external advertisement revenue, and the company does not, and will never, sell or otherwise provide user data for any commercial purpose, directly or indirectly. X2 suggests this is an essential standard for any emotional support AI service provider. X2 leadership believes that the service itself, and not the possibility of providing data about a particular user, must be the source of value generated in this business model.

In some instances in which X2’s privacy policy allows, X2 may seek to use random samples of de-identified user data that is reported in aggregate solely for research purposes; this is made transparent so that users can decide if they are willing to participate. Notably, any user may later delete his/her data from the system at any time. If users opt-in, results are reported in aggregate. For AI coaches customized for research institutions, such as Duke University, Palo Alto University, Rochester Institute of Research, and Nemours Children's Hospital, these institutions and their Institutional Review Boards (IRBs) administer research participation consent.

In addition to the Terms of Use and Privacy Policy, all users receive a disclaimer asking if they understand that the AI coach is not a person and is not a crisis response service (along with direct contact information for crisis services). X2 developed the AI coaching dialog for crisis evaluation in collaboration with crisis management professionals. If users indicate that they may be in crisis during their chat, they are again provided with direct contact information for crisis services in their area/region and encouraged to seek this support. For X2’s business-to-business (B2B) services, including Employee Assistance Programs (EAPs), the company offers a network of thousands of 24/7 professional crisis management counselors. Should a user affirm the need for crisis management services at any time during his/her chat, this integration makes it possible for a human crisis response professional to take the place of the AI coach. Importantly, any instances of crisis switchover are made fully transparent to the user.

Accountability

The responsibility of ensuring ethical AI rests with the people creating it. Accordingly, X2 prioritizes ongoing processes for monitoring and assessing decisions and actions. The policy, content, research, and technical leaders meet regularly to evaluate best practices and research priorities, as well as develop new strategies for mitigating potential misuse of AI and user data.

The medical ethical board, which meets semi-annually, is the most important source of ongoing external feedback. X2 leadership convenes focused discussions with established mental health and technology scholars and experts to advance the companies understanding of the complex ethical questions that guide the work. In between these meetings, X2 maintains active discussion with the advisory board members and seeks their input when any questions or concerns arise. Each member of the medical ethical board has acted as a content creator, user, or provided guidance who directly contributed to the system development, making his/her insights especially impactful. In addition to participating in semi-annual meetings, the role of each board member includes the following:

- Understand and achieve alignment around X2’s mission, services, and core values.

- Advance X2’s understanding of complex ethical questions that guide our work.

- Deliver guidance when X2 faces ethical dilemmas or concerns.

The focus on accountability extends beyond X2 to include partner organizations. Because X2’s services are designed to be highly customizable and widely accessible, the company works with clients to determine which communication platform(s) best serve users such as SMS (text messaging), Facebook Messenger, Signal, Slack, and more. While the chat interfaces X2 works with include HIPAA- and GDPR-compliant offerings, X2 also prioritizes accessibility through everyday channels available to users worldwide. X2 works closely with clients alongside partner organizations to assess and mitigate any potential risks to users.

Team expertise and training

X2’s team expertise and training bolster the ethical commitments at every level of engagement. X2’s staff and advisors include licensed and experienced psychologists (PhD/PsyD/LLP), accredited psychiatrists (MD), board-licensed professional counselors, AI and machine learning experts, and superusers. When new team members join X2, they participate in the onboarding curriculum, and all team members take part in ongoing discussions concerning AI ethics and security requirements (apart from regular employee background checks). X2 provides all its employees with career development opportunities such as continuing education courses for mental health professionals and coverage for registration fees for symposiums that enhance product knowledge and expertise - a policy that aligns with recommendations by both the APA and the Association for Computing Machinery [3,9]. Following the recommendations by the White House stipulating that AI ethics should be clearly linked to practical efforts and outcomes, training at X2 is augmented with technical tools and methods for actively working to prevent unacceptable outcomes [7]. For instance, X2 provides training to team members, research partners, and clients to white-label and tailor each unique version of the core chatbot (known as “Tess”) through their proprietary HIPAA-compliant platform. The knowledge and experience of team members ensure that ethics are never compromised by the technology used.

Monitoring and Minimizing Bias

One key benefit of integrating AI into mental health practices is to better provide a safe and non-judgmental space for people to explore sensitive concerns. Many people experience shame and marginalization due to a diagnosis and go on to describe the consequences of stigma as more burdensome than those of the condition itself [10]. While AI in this context may have the capacity to help alleviate bias and stigma, it is also the case that bias can arise in unanticipated ways within machine learning systems. X2 aims to prevent this by limiting the role of AI and carefully maintaining human oversight of it. Bias is further reduced, thanks to the globally diverse user base across Africa, Asia, Australia, Europe, and South America through X2 AI Inc. and X2 Foundation [11]. The system is trained with evidence-based interventions such as those rooted in Cognitive Behavioral Therapy, Motivational Interviewing, Mindfulness-Based Therapy, and more that are drafted by mental health professionals who are highly informed about bias and stigma. By strategically applying machine learning features to this closely managed content, X2 enables AI to develop appropriately through interactions with users.

X2 also globally monitors the AI system for any indication of absent or inadequate content areas and for any instances of language or emotion error. By gathering and monitoring de-identified training data from an array of interactions, not only does the AI improve, but also the team learns how to more effectively anticipate and prevent bias and stigma in content creation. To minimize bias brought in through this process, X2 employs a diverse team of core staff and external content developers.

Additionally, X2 frequently invites users to provide direct input on services in a number of ways. The AI coaches are designed to request feedback every few conversations, much in the way a human coach or therapist does. Feedback questions are structured to gather qualitative feedback such as “Is there anything I can do to better support you?” and more quantitative questions to evaluate net promoter score and user satisfaction. The team consolidates this feedback into categories that are regularly incorporated into core team discussions. The review process includes analyzing feedback for any indications that users may be experiencing or be subject to bias or stigma of any kind within X2 services. Occasionally, this review process overturns some anticipated areas of bias or exclusion. For instance, X2 expected that older adult users might be less inclined to engage with, or benefit from, an AI coach, but preliminary findings from a trial with a major senior care provider indicated that interactions for four weeks increased social connectedness in older adults by 55.7%, as measured by the DSSI (public results forthcoming).

“AI on a Leash”

AI systems require thorough training from humans on how to best support people. X2 believes that the role of AI should be restricted to ensure that human expertise appropriately guides all AI interactions and decisions. While the X2 system might provide insights into the effectiveness of certain wording or emotion identification, any adaptation is carefully reviewed by our experts before it is implemented. In general, any word or sentence that the AI coach responds with is pre-scripted and approved by professionals with experience on the subject matter. X2’s staff of mental health professionals and computer scientists work collaboratively at every step in the design and maintenance of the system.

X2 applies this same strategy of keeping “AI on a leash” when creating AI coaches and training them on which emotions, topics, psychological modalities, and interventions are applicable in what contexts. Without exception, X2 works closely with experienced clinical psychologists to create all of the emotional support content. Similarly, to form the personality of the primary English language AI coach (Tess), the APA’s ethical principles guided the selection of character traits [3]. X2 used this framework to develop the AI coach’s “clinical style” or, in other words, elements such as tone, boundary setting, crisis intervention procedures, and the appropriate amount of “self-disclosure” (for instance, when asking a user to describe a relaxing activity they enjoy, Tess might indicate enjoying surfing the web). X2 worked closely with industry experts to achieve a character template that is highly customizable and responsive to the parameters set.

Ensuring User Inclusivity

One key benefit of the customizability of the X2 system is that existing content can be leveraged for each new iteration, making it easier to incorporate appropriate cultural nuances for users worldwide. For every AI coach that X2 creates, the team begins by identifying user needs and then developing a set of core modules. These modules include an introduction, brief intake, crisis support script, and relevant interventions categorized by psychological modality. These are then reviewed and revised by subject matter experts to improve reliability and applicability with regard to language, quality of support, local resources, and similar factors.

X2 currently offers AI coaching services in Arabic, Dutch, English, Japanese, and Spanish, and plans to expand its scope of service delivery by adding additional language options. X2 considers many factors during the translation process and continually updates best practices as the range of languages and total user count increases. To give one example, because Spanish applies grammatical gender, the Spanish language AI coach must request the user’s gender preference in the first conversation. While professional and user feedback indicates that this is an appropriate option at this time, X2 continues to work toward providing more inclusive and sensitive support to users whose gender may not be among the options stated. This is one topic among many that are discussed during weekly content strategy sessions in which the team examines metrics to inform content enhancements.

When considering the role of AI coach in emotional support, it is important to recognize unique preferences across different cultures and groups of users [12]. X2 has internally analyzed and identified some of these differences based on user-AI interactions and direct user feedback [13]. For example, Spanish-speaking users from Latin American countries have shown a tendency to reply with longer descriptions and more variety of words and phrases than English-speaking users from the United States, Australia, and Europe. Likewise, qualitative and subjective feedbacks from adolescent and student users have expressed a preference for a speedy response from the AI coach, whereas those over 50 years of age have expressed a preference for a more natural response speed that mimics a person-to-person message exchange.

X2 also evaluates AI coach word choice, tone, and intervention selection to ensure that the level of education does not limit user access. For instance, the Monterey County Health Department requested minor script edits featuring more general use language to improve user connection with the AI coach. X2 analyzed feedback, including drop-off and error rates, to precisely identify which words and phrases to edit. These findings enabled greater inclusivity and also helped to develop new conversation modules. Community feedback directly informed of pressing areas of concern for Monterey County residents at the time, including political anxiety, fear of deportation, and substance abuse.

Research and development

Strong emphasis on research and development is necessary to provide safe, confidential, and secure emotional support through AI. X2 allocates approximately one-third of time and resources to research efforts focused on evaluating the feasibility and efficacy of the AI system. This aligns with recommendations by the US and UK governments and the Partnership on AI (an international technology industry consortium) for international engagement with industry experts, organizations, and academia to exchange information and collaborate on AI research and development [7,14]. X2’s primary research categories are as follows:

1. **Feasibility studies** to evaluate the use of AI both as a stand-alone source of emotional support and as an adjunct to existing programs and treatment solutions. This includes partnerships with the Centre for Aging and Brain Health Innovation (CABHI) and Saint Elizabeth Health Care to display how Tess is being used to support 9,000+ employees and 10,000+ patients with text-based and voice-enabled emotional support [15]. Furthermore, IBH Corp.’s EAP case study revealed that Tess interactions support employees and cut organizational costs with 10,000 messages exchanged pre-launch, which would have cost their staff 20,000 minutes, an equivalent to two months of work by one full-time equivalent when assuming an eight-hour workday, which at $65 per hour would be equivalent to $21,667 [16]. Additional feasibility studies are underway with a major senior care provider and Universidad de Palermo.

2. **Generalizability and scalability studies** to evaluate the use of AI as a source of accessible, affordable, and inclusive emotional support to all people on a global scale. Examples include Duke University’s IRB-approved single-case experimental design pilot study to expand access to perinatal depression treatment in Kenya through Tess, and the Universidad Adventista del Plata’s randomized controlled trial that revealed that Tess interactions led to significantly reduced symptoms of depression by 28%, as measured by the Patient Health Questionnaire (PHQ)-9 (p=0.02), and anxiety by 18%, as measured by the General Anxiety Disorder 7-item scale (GAD-7) (p=0.04) [17]. Through a grant awarded by the Baycrest CABHI, 20,000 older adults are being given access and are able to talk to Tess through Facebook Messenger or Google Home [15]. The ongoing generalizability and scalability studies include partnerships with a Nigerian Federal Psychiatric Hospital, a group of universities in Singapore, and Erasmus University in the Netherlands.

3. **Efficacy studies** to evaluate the use of AI as a partner to professionals, including specialized emotional support areas/topics or coaching programs, as well as clinical treatment and symptom identification. This includes the randomized controlled trial with Northwestern University, which revealed that Tess interactions led to significantly reduced symptoms of depression by 13%, as measured by the PHQ-9 (p=0.03), and anxiety by 18%, as measured by the GAD-7 (p=0.02) [13]. Research with Nemours Children's Hospital revealed Tess to be a beneficial adjunct to pediatric care as adolescent patients experienced positive progress toward their goals 81% of the time and reported usefulness ratings 96% of the time [18]. Ongoing and upcoming efficacy studies will be completed through research partners such as Palo Alto University, Stanford University, CHADIS (Comprehensive Health and Decision Information System), and Patients-Centered Outcomes Research Institute.

These researchers’ efforts consistently inform content development. X2’s AI technology is not currently designed to deliver emotional prediction or interpretation with authority, such as diagnosis. Instead, it is designed to respond to users with advice and interactions that produce a helpful effect. As noted above, any content expansion or system development that extends beyond the core team’s expertise is completed in collaboration with industry experts. These experts include clinical, ethical, and technical advisors, content developers, and research collaborators. By guiding content creation, AI personality development, privacy and security policies, research, and more, many subject-matter experts have already contributed to the ethical improvement of X2’s services.

## Discussion

Efforts to develop ethical standards for AI are not always successful, as made apparent by Google recently facing shutdown of their external advisory council on AI ethics in 2019 [19]. First and foremost, Google drew a strong critique for the selection of council members [20]. This highlights the advantage of incorporating technology ethics alongside mental health professional ethics in the code. The commitment to harm reduction, to which each of X2’s medical ethical board members is accountable, provides a strong and clear basis for selecting external expert guidance. Google received further criticism for planning insufficient council interaction for a company operating at its scale. While the needs of a startup differ from that of one of the world’s largest companies, this critique nevertheless illustrates why X2’s effort toward ethical operations incorporates both formal meetings and ongoing discussions between meetings. X2’s code reflects what was discovered in creating it: the importance of linking ethical considerations to concrete practices that are rooted in sustained interactivity and collaboration within and beyond the company.

Notably, an industry-led ethical code is not a rejection of the possibility of expanded regulatory oversight. Instead, it equally lays the groundwork to inform future regulation and industry-led efforts alike. Simply put, the stronger the alignment between members of this industry on ethical standards and procedures, the more likely it becomes that AI can provide emotional support for as many people as possible. With this technical report, we hope to set the bar high for ethical standards within our industry (niche).

## Conclusions

The guiding principles and practices described provide an ethical code template that can assist startups and other organizations in creating effective emotional AI solutions while ensuring the safety and privacy of clients and users. AI coaches offer affordable and scalable on-demand care, and a growing body of research demonstrates its impact and efficacy in helping people to feel better. Industry-wide uptake of a process-driven ethical code will increase the accessibility of these services and facilitate wider access to support worldwide.
